# Species Diversity, Habitat Distribution, and Blood Meal Analysis of Haematophagous Dipterans Collected by CDC-UV Light Traps in the Dominican Republic

**DOI:** 10.3390/pathogens11070714

**Published:** 2022-06-21

**Authors:** Mikel Alexander González, Daniel Bravo-Barriga, María Altagracia Rodríguez-Sosa, Juan Rueda, Eva Frontera, Pedro María Alarcón-Elbal

**Affiliations:** 1 Institute of Tropical Medicine and Global Health (IMTSAG), Universidad Iberoamericana (UNIBE), Santo Domingo 10203, Dominican Republic; mikel_alexander86@hotmail.com (M.A.G.); a.investigacion1@uafam.edu.do (M.A.R.-S.); juan.rueda@uv.es (J.R.); pedro.alarcon@uv.es (P.M.A.-E.); 2 Applied Zoology and Animal Conservation Group, University of the Balearic Islands (UIB), 07122 Palma de Mallorca, Spain; 3 Parasitology and Parasitic Diseases, Animal Health Department, Veterinary Faculty, University of Extremadura (Uex), 10003 Cáceres, Spain; frontera@unex.es; 4 Cavanilles Institute for Biodiversity and Evolutionary Biology (ICBiBE), University of Valencia, 46980 Paterna, Spain

**Keywords:** *Culicoides*, mosquitoes, blood meals, DNA barcoding, new records, the cytochrome *c* oxidase subunit 1 (COI)

## Abstract

Haematophagous insects cause major economic losses by both direct damage and the transmission of pathogens. However, the biting Diptera species in the Caribbean region have been poorly documented. During 2021, CDC downdraft suction traps with UV light were employed to assess both the species occurrence and blood meal sources across three different habitats in the Dominican Republic. Eighteen species of mosquitoes (*n* = 274), six species of *Culicoides* (*n* = 803), two black fly species (*n* = 2), and one species of muscid fly (*n* = 25) were identified at species-level by morphology and/or molecular phylogenetic approaches based on the mitochondrial cytochrome *c* oxidase subunit 1 (COI). Engorged mosquito (*n* = 5) and *Culicoides* (*n* = 28) females showed host preferences derived exclusively from mammals (cows and pigs), except *Culex* species containing the blood of chickens. Our study provides new records of the Diptera Dominican catalogue (*Culex salinarius* for the Greater Antilles, *Culicoides jamaicensis* for Hispaniola, and *Culicoides haitiensis* and *Culicoides borinqueni* for the Dominican Republic), the first available COI DNA sequences of different Diptera in the GenBank, some pictures of diagnostic features of closely related specimens, spatial distribution across the habitats studied, and new insights on their feeding preferences in the Caribbean region.

## 1. Introduction

Vector-borne diseases remain a major threat for Caribbean health agencies and stakeholders. In the geographical context, favourable climatic conditions allow a practically uninterrupted transmission pattern throughout the year, becoming a great impediment to social and economic development [1]. Although the interest of these diseases has grown due to the increase in some arbovirus outbreaks [2], there is scant information on vectors in islands such as Hispaniola, except for immature stages of mosquitoes (Diptera: Culicidae), which have been studied extensively in recent years [3,4]. Nonetheless, knowledge of other relevant haematophagous Diptera of medical and veterinary interest is still very scarce and needs to be updated with entomological studies.

Culicids are considered the most relevant arthropod family in human and animal health worldwide [5], and in the insular Caribbean [1,6]. In the Greater Antilles, some synanthropic and well-distributed mosquito species, such as *Aedes aegypti* (Linnaeus, 1762), *Aedes albopictus* (Skuse, 1894), and *Culex quinquefasciatus* (Say, 1823), are the main species responsible for arbovirus transmission [1,7]. The recent finding of *Aedes vittatus* (Bigot, 1861), a new potential vector species of yellow fever, Zika, chikungunya, and dengue virus [8], in the Americas has recently gained considerable attention. It was first recorded in 2020 in the Dominican Republic and Cuba [9,10], and its local distribution is still unknown because it has only been spotted in specific locations.

Tiny biting midges of the genus *Culicoides* (Diptera: Ceratopogonidae) are responsible for the transmission of both human and veterinary vector-borne diseases in the insular Caribbean. *Culicoides furens* (Poey, 1853) and *Culicoides barbosai* (Wirth & Blanton, 1956) were reported as the main vectors of *Mansonella ozzardi* (Mansoni, 1897) in Haiti, but this filarial nematode has never been reported in the neighbouring Dominican Republic [11,12,13]. The Oropouche virus (OROV) is also an emerging zoonotic disease transmitted by biting midges; it was recently detected in the plasma of children in schools of Haiti [14]. Bluetongue virus (BTV) is considered the most important veterinary disease transmitted by *Culicoides*, mainly in sheep and some wildlife species. In the Dominican Republic, serotypes BTV-4, BTV-6, and BTV-8 were detected by virus isolation several times at the end of the 20th century [15]. Despite the epidemiological importance of biting haematophagous Diptera, their study in the Dominican Republic has been ignored for decades.

The identification of blood meal sources of arthropod vector species contributes to the understanding of host–vector–pathogen interactions, and provides important insights into the dynamics of viral, parasitic, or bacterial transmission, allowing public health authorities to design and implement efficient strategies for vector control [16]. Molecular markers, i.e., the cytochrome *b* (*cyt-b*) and cytochrome *c* oxidase subunit 1 (COI) are widely used to accurately address host range preferences and to replace animal-baited traps, landing experiments, and serological methods such as the precipitin test or enzyme-linked immunosorbent assays (ELISA), which present severe limitations [17]. COI-based DNA barcoding is also a useful tool to complement taxonomy-based identification of mosquito species. However, these molecular techniques have not been widely implemented in many low-and middle-income Caribbean countries. In addition, the accurate identification of arthropod vectors is challenging due to the absence of updated keys, the damage of the external characters due to improper specimen handling or storage, and overall, due to the lack of well-trained staff in medical-veterinary entomology, a specialized discipline that has been regressing in the region for at least two decades [18].

The above-mentioned reasons might explain the low number of studies on the diversity of arthropod vector species and host interactions from the Caribbean region. Therefore, based on traditional morphological methods accompanied by DNA barcoding, the present study aimed to provide accurate information on the fauna of nocturnal/crepuscular haematophagous dipterans, and to assess their host blood meal sources in Jarabacoa, a touristic mountainous area of the Dominican Republic.

## 2. Results

A total of 1104 haematophagous dipteran specimens of at least 27 species of four families: 18 species of Culicidae, including two unknown species (*Culex* sp. and *Uranotaenia* sp.); six species of Ceratopogonidae; two species of Simuliidae; and one of Muscidae were captured and identified at species-level. COI barcodes were generated for 12 mosquito species and four *Culicoides* species; six of them (three mosquitoes and three *Culicoides*) were not previously available in public sequence databases. Differences in spatial distribution across the three habitats were significant for biting midges. Host DNA sequences were generated from 33 blood-engorged specimens of six and three species of mosquitoes and *Culicoides*, respectively.

### 2.1. Species Composition Based on Morphological Features

**Figure 1:**
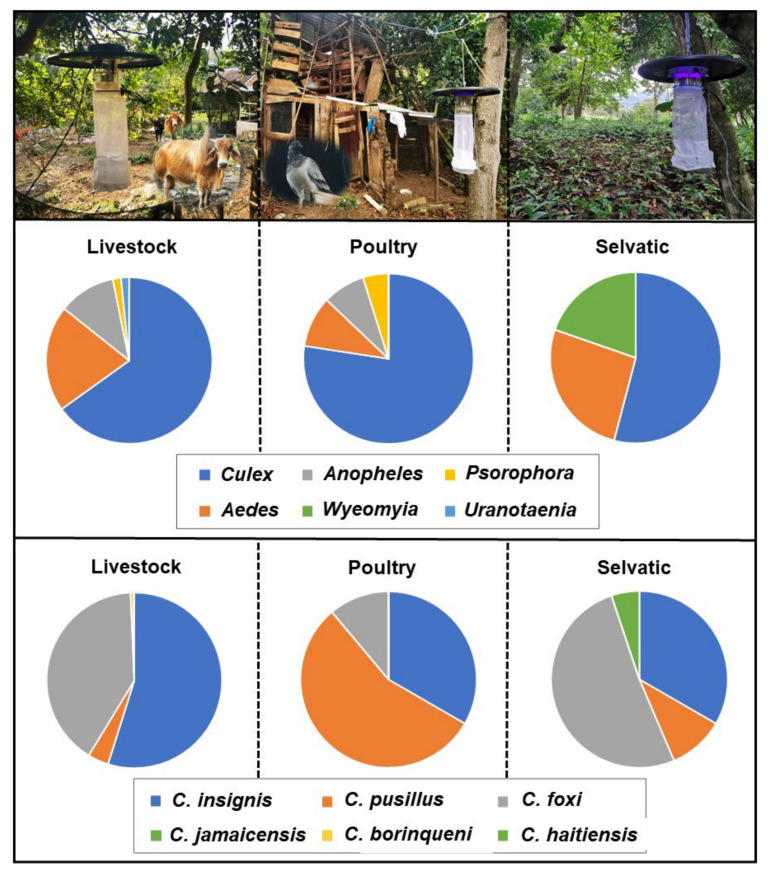
Schematic representation of the distribution of mosquitoes (above, by genera) and *Culicoides* (below, by species) across the three habitats sampled in Jarabacoa (Dominican Republic) by CDC UV light traps.

A total of 263 mosquito specimens of 11 species were identified morphologically. The following species were recorded in decreasing order of abundance: *Cx. quinquefasciatus* (145 females, 32 males), *Aedes scapularis* (Rondani, 1848) (22 females), *Culex secutor* Theobald, 1901 (13 females, 4 males), *Wyeomyia mitchellii* (Theobald, 1905) (10 females, 1 male), *Anopheles albimanus* Weidemann, 1820 (11 females), *Anopheles crucians* Wiedemann, 1828 (7 females), *Ae. vittatus* (4 females, 2 males), *Psorophora confinnis* (Lynch Arribálzaga, 1891) (4 females), *Ae. albopictus* (3 females), *Uranotaenia sapphirina* (Osten Sacken, 1868) (2 females, 1 male), and *Anopheles grabhamii* Theobald, 1901 (2 females). In addition, 11 specimens could be identified to genus-level: *Culex* spp. (8 females), *Aedes* sp. (1 female), *Wyeomyia* sp. (1 female), and *Uranotaenia* sp. (1 female) (Figure 1).

**Figure 2:**
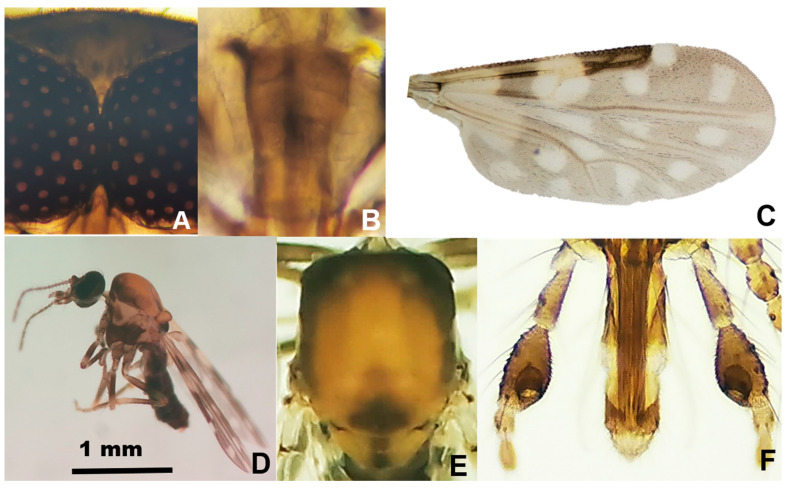
*Culicoides haitiensis*: (**A**) eyes, (**B**) cibarium, (**C**) wing, (**D**) general aspect, (**E**) *scutum* and *scutellum*, and (**F**) palps.

**Figure 3:**
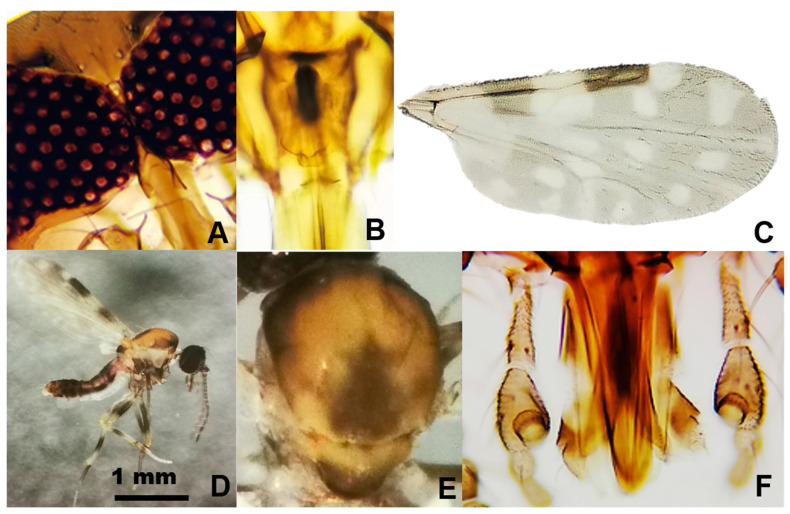
*Culicoides borinqueni*: (**A**) eyes, (**B**) cibarial armature, (**C**) wing, (**D**) general aspect, (**E**) *scutum* and *scutellum*, and (**F**) palps.

**Figure 4:**
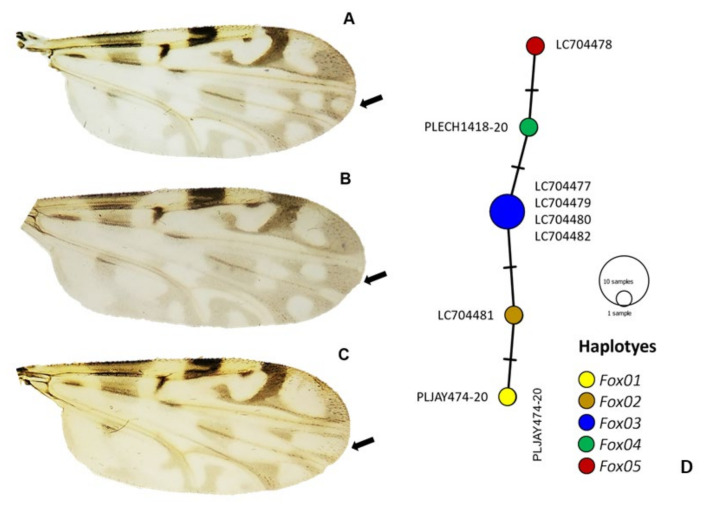
*Culicoides foxi* morphospecies and haplotypes. (**A**) The typical morphospecies with a pale spot on m1; (**B**) the morphospecies without a spot on m1; and (**C**) the morphospecies with both spots on m1 joined. The arrows indicate the position of the spots. (**D**) Median joining network of the haplotypes. Note: each circle represents a haplotype.

Among the 803 specimens of *Culicoides*, six species were reported in the following order of abundance: *Culicoides insignis* Lutz, 1913 (392 females, 32 males), *Culicoides foxi* (Ortiz, 1950) (300 females, 21 males), *Culicoides pusillus* Lutz, 1913 (36 females, 15 males), *Culicoides borinqueni* Fox and Hoffman, 1944 (4 females), *Culicoides jamaicensis* (Edwards, 1922) (2 females), and *Culicoides haitiensis* Delecolle, Raccurt, and Rebholtz, 1986 (1 female) (Figure 1). *Culicoides haitiensis* and *C. borinqueni* were recorded for the first time for the Dominican Republic, whereas *C. jamaicensis* was reported for the first time for Hispaniola. Illustrated diagnostic characters of *C. haitiensis* and *C. borinqueni* females are provided for the accurate separation of both sibling species (Figure 2 and Figure 3). Fresh specimens can be easily separated under the stereomicroscope according to the wing pattern and pale spot of the *scutellum.* Mounted specimens can be separated based on the presence of cibarial armature, which is only present in *C. borinqueni.* Specimens of *C. foxi* were allocated to three different morphotypes according to their wing pattern: (i) typical morphospecies with a spot on the distal part of m1 (*n* = 290), (ii) morphospecies without a spot on m1 (*n* = 27), and (iii) morphospecies with both spots on m1 joined (*n* = 3) (Figure 4A).

Other collected haematophagous insects, the black fly species (Diptera: Simuliidae) *Simulium quadrivittatum* Loew, 1862 (*n* = 1), and *Simulium haematopotum* Malloch, 1914 (*n* = 1), and *Haematobia irritans* (Linnaeus, 1758) (Diptera: Muscidae) (*n* = 25) were also recorded by suction traps. No sand flies (Diptera: Psychodidae) were collected.

### 2.2. COI-Based DNA Barcodes and Phylogenetic Analysis

**Figure 5:**
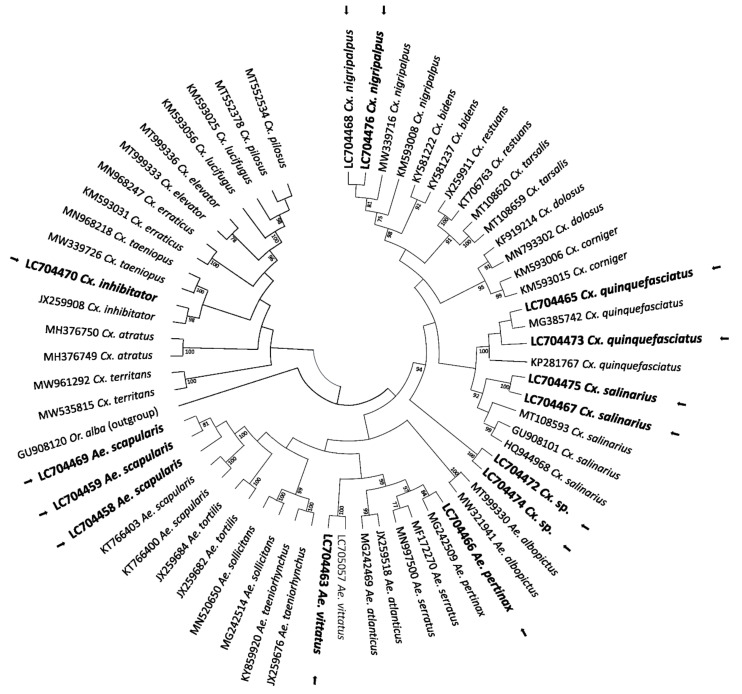
Evolutionary history of *Culex* and *Aedes* genus samples. The tree with the highest log likelihood (−2981.46) is shown. Initial tree(s) for the heuristic search were obtained automatically by applying the Neighbor-Join and BioNJ algorithms to a matrix of pairwise distances estimated using the maximum composite likelihood (MCL) approach, and then selecting the topology with superior log likelihood value. A discrete gamma distribution was used to model evolutionary rate differences among sites (five categories; +G, parameter = 0.3796). The rate variation model allowed for some sites to be evolutionarily invariable (+I, 30.86% sites). This analysis involved 63 nucleotide sequences. All positions containing gaps and missing data were eliminated (complete deletion option). There were 405 positions in the final dataset. At specific branch nodes, bootstrap values >75% in 1000 repetitions are indicated. *Orthopodomyia alba* Baker, 1936 (GU908120) was used as an outgroup sequence. The sequences of our study are presented in bold with an arrow.

**Figure 6:**
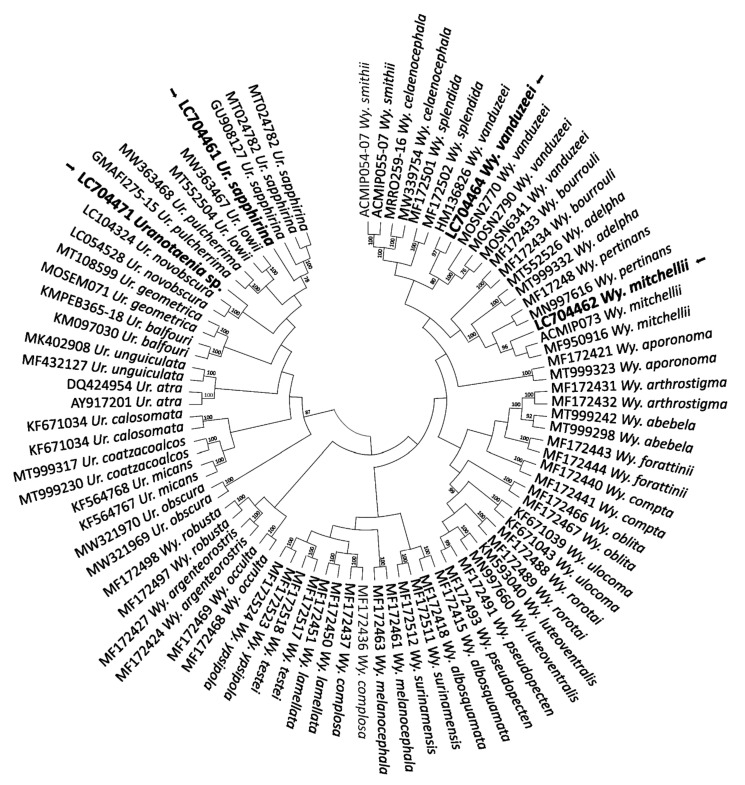
Evolutionary history of *Uranotaenia* and *Wyeomyia* samples. The tree with the highest log likelihood (−4386.72) is shown. Initial tree(s) for the heuristic search were obtained automatically by applying the Neighbor-Join and BioNJ algorithms to a matrix of pairwise distances estimated using the maximum composite likelihood (MCL) approach, and then selecting the topology with superior log likelihood value. A discrete gamma distribution was used to model evolutionary rate differences among sites (five categories; +G, parameter = 0.4512). The rate variation model allowed for some sites to be evolutionarily invariable (+I, 28.79% sites). This analysis involved 87 nucleotide sequences. All positions containing gaps and missing data were eliminated (complete deletion option). There was a total of 356 positions in the final dataset. At specific branch nodes, bootstrap values >75% of 1000 repetitions are indicated. Our sequences are shown in bold with an arrow.

For mosquitoes, 18 COI DNA barcode sequences were analysed for phylogenetic reconstruction by comparing them with barcodes from public databases. The ML tree allowed to confirm the identification of 10 mosquito species at species-level, plus two at genus-level: *Ae. scapularis* (LC704458, LC704459, LC704469; *n* = 3), *Ae. vittatus* (LC704463; *n* = 1), *Aedes pertinax* Grabham, 1906 (LC704466; *n* = 1), *Culex nigripalpus* Theobald, 1901 (LC704468, LC704476; *n* = 2), *Cx. quinquefasciatus* (LC704465, LC704473; *n* = 2), *Culex inhibitator* (Dyar and Knab, 1906 (LC704470; *n* = 1), and *Culex salinarius* Coquillett, 1904 (LC704467, LC704475; *n* = 2), which represents the first report from the Greater Antilles. On the other hand, the samples referred to as *Culex* sp. (LC704472, LC704474; *n* = 2) could not be identified to the species-level based on homology and phylogenetic analysis (Figure 5). The nucleotide sequences of the *Uranotaenia* and *Wyeomyia* genera revealed an overall congruent topology with a consistent placement of the obtained sequences in the cluster with *Wy. mitchellii* (LC704462, *n* = 1), *Wyeomyia vanduzeei* Dyar and Knab, 1906 (LC704464, *n* = 1), and *Ur. sapphirina* (LC704461, *n* = 1). However, regardless of the strategy used for phylogenetic reconstruction, the sample *Uranotaenia* sp. (LC704471, *n* = 1) could not be identified to the species-level (Figure 6). The sequence homology value of the sample was <93%.

Regarding *Culicoides*, there were no available sequences for *C. jamaicensis*, *C. haitiensis*, and *C. borinqueni*; thus, our barcodes represent the first GenBank records of these species, and have been summited under the following accession numbers: *C. jamaicensis* (LC704483–LC704485), *C. haitiensis* (LC704939), and *C. borinqueni* (LC704940). We also obtained a total of six sequences of the three different morphotypes of *C. foxi* (Figure 4A–C). The analysis of pairwise distance values indicated that *cox*1 mtDNA sequences are very similar to one another, with a genetic diversity distance of 0 or close to 0 between Costa Rica and Dominican Republic samples (Table S1 in Supplementary File). Therefore, there are no phenotypic differences with the COI marker used. The Hd and Pi for all the *C. foxi* sequences analysed were 0.786 ± 0.022 and 0.002 ± < 0.000, respectively, and for the Dominican Republic samples, the values were 0.600 ± 0.046 and <0.000 ± < 0.000, respectively. A total of five haplotypes (Fox1–Fox5) were detected from eight aligned sequences (Figure 4D), although a larger study would be necessary in view of the lower intraspecific diversity. The most prevalent haplotype was Fox3, which represented 50% (4/8) of all *C. foxi* sequences (Figure 4D). Sequences from each haplotype were deposited in GenBank under accession numbers LC704477–LC704482.

On the other hand, the identification of *H. irritans* was confirmed molecularly, and showed 100% homology with public sequences from GenBank (e.g., accession number: KM669714).

### 2.3. Spatial Distribution

The abundance of mosquitoes did not reveal significant differences among the three habitats (KW = 2.069, degrees of freedom [df] = 2, *p* = 0.355). The species richness was higher in the sylvatic habitats (*n* = 16) compared to livestock (*n* = 11) and poultry (*n* = 10) habitats. *Culex quinquefasciatus* was the most common species, found in 21 out of the 30 sampled sites (Figure 1). The newly introduced species *Ae. vittatus* was spotted in four sites. *Culicoides* were significantly more abundant in livestock farms compared with both poultry and sylvatic habitats (KW = 17.818, df = 2, *p* ≤ 0.05). Livestock habitats comprised higher number of species (*n* = 5) compared to sylvatic (*n* = 4) and poultry (*n* = 3) habitats. *Culicoides insignis* was the most frequent species, appearing in 18 out of the 30 sites (Figure 1).

### 2.4. Blood Meal Analysis

DNA from host blood meal sources was amplified successfully in 100% of the mosquito specimens analysed. The study of haematic preferences indicated a diet based mainly on avian and mammalian hosts. *Culex* species fed on chicken, whereas *Aedes* spp. and *Ps. confinnis* fed on pigs (Table 1). The overall amplification success was 65.1% in biting midges, being notably lower in *C. pusillus* compared with *C. insignis*. The blood meals of these two species and of *C. foxi* were derived exclusively from cows (Table 1).

## 3. Discussion

This work is pioneering in applying molecular tools (COI-based DNA barcoding) for the identification of mosquitoes, *Culicoides* biting species, and their host blood meal sources in the insular Caribbean. Previously, DNA barcoding had only been used to confirm the first record of *Ae. vittatus* in the Americas [10]. To our knowledge, the last study on host-derived blood meals was carried out in the 1980s in Hispaniola during an eastern equine encephalitis outbreak; the investigation employed precipitin tests [19].

Approximately 52 species of living mosquitoes have been recorded from Hispaniola [3,4,10,20,21,22]. However, most of these records were based on the identification of mosquitoes in the III–IV larval stage. Our identification effort revealed the presence of at least 16 species. However, two other species (*Culex* sp. and *Uranotaenia* sp.) require further confirmation because COI barcoding is dependent on the availability of representative sequences for comparison, and this approach fails when insufficient reference sequences are deposited in databases [23,24]. In addition, there is a lack of molecular and taxonomist professionals (and, therefore, a lack of identification keys), which also leads to misidentifications, making the interpretation of COI sequences problematic and inaccurate [25]. This fact is aggravated because *Culex* and *Aedes* species recognition is mainly based on adult morphology, but the absence and overlap of morphological characters have often been identified as factors that lead to the misidentification of these mosquito species [26]. Nevertheless, COI-based DNA barcoding was a useful tool to identify the remaining species, and contributed to display the phylogenetic relationships among the species. It was also useful for the determination of the blood-fed *Culex* specimens that were initially classified as *Cx. quinquefasciatus* based on morphological features, but, subsequently, were characterised molecularly into *Cx. nigripalpus*. This is not a trivial matter, as blood-engorged specimens show modification of the entire body, and scales and bristles of terga rub off or are distorted. For all these reasons, COI-based DNA barcoding should be used for accurate and precise identification of sibling species, species complexes, damaged specimens, and also blood-fed specimens.

Exotic/invasive species are among the primary threats to biodiversity in Hispaniola because they can cause environmental and economic damage [27], as well as negatively impact human and animal health through disease transmission [28]. Our study also expands the distribution of *Ae. vittatus* to other sites, including urban and rural settings, increasing the spatial distribution of this newly discovered exotic species. It is interesting to note that this species was trapped by UV-suction traps, which is not a particularly useful tool to collect most diurnally active *Aedes* mosquitoes, given that they show little positive attraction to light traps [29]. However, *Ae. vittatus* seem to be an exception because they can also be collected sporadically in non-baited light traps [10].

Regarding the finding of *Cx. salinarius*, this species has been found in The Bahamas, Bermuda, Canada, Mexico, and the United States [30]; therefore, this is the first time it has been reported in the Greater Antilles. This species closely resembles both the adult and immature stages of *Cx. quinquefasciatus*, and may be found in cohabitation. It is plausible to think that *Cx. salinarius* has been misidentified as *Cx. quinquefasciatus* in previous studies undertaken in the country. This culicine species has been incriminated as a potential bridge vector of several encephalitis viruses, such as West Nile virus, St. Louis encephalitis virus, and eastern equine encephalitis virus, among others [31]. The most relevant mosquito species in relation to the transmission of equine encephalitis in the Dominican Republic is *Cx. quinquefasciatus*, due to its feeding habits, and also to its great synanthropy and ubiquity [32], as seen in our study. However, *Cx. salinarius* should also be taken into account in vector control programmes from now on, especially in rural areas of the country, such as the municipality of Jarabacoa.

It should be pointed out that the type of collection method employed (i.e., CDC UV light suction traps) is not considered an appropriate tool for the collection of some mosquito species, particularly diurnal species, and thus, the checklist recorded in this manuscript might represent only part of the mosquito diversity present in the study area.

Only 12 species of extant *Culicoides* have been recorded in Haiti, and four in the Dominican Republic in the early 1990s [19,33,34,35]. However, this list requires further revision because Mitchell [19] recorded a single specimen of *Culicoides obsoletus* (Meigen, 1818) in Haiti, a species only found in the Palearctic region. With the incorporation of *C. haitiensis*, *C. borinqueni*, and *C. jamaicensis*, the number of living species in the Dominican Republic and Hispaniola has increased to seven and 13, respectively. Our study also provides the first pictures of the thorax of *C. haitiensis*, which possesses a characteristic dark spot on the *scutum* and *scutellum* that was not recorded in the original descriptions of Delecolle et al. [34] because the specimens were damaged. These characteristics allow unequivocal separation of this species from other related species without the need to mount specimens. We have also provided additional pictures of other important diagnostic characteristics, and the first COI barcoding sequences of the three mentioned *Culicoides* species. The molecular information provided for *C. jamaicensis* shed light on the unresolved question about the origin of this species in the Americas. Specifically, Meiswinkel et al. [36] postulated that *C. jamaicensis* and *Culicoides paolae* (Boorman, 1996) are morphologically similar, suggesting the possibility that the former was introduced into the Mediterranean Region at the time of Columbus. A deep molecular analysis coupled with a detailed morphometric study might help to resolve this mystery. It also interesting to note that, most likely, the *C. foxi* phenotypic differences respond to intraspecific variations within the same species as a consequence of different environmental conditions in breeding sites, or other unknown reasons. The number of haplotypes found suggests a relatively high diversity compared with other *Culicoides* species [37].

The analysis of field-collected engorged females through PCR amplification of host DNA present in a blood meal is a valuable method to determine host use of many blood-feeding arthropods [17]. Unfortunately, in our study, blood-engorged specimens were very scarce; hence, we could only draw preliminary conclusions. *Culex quinquefasciatus* was quite abundant, particularly in poultry shelters. *Culex* spp. are known to feed on a wide range of vertebrate species, including avian and mammalian hosts [38]. *Culex quinquefasciatus* has shown a wide host preference range in the insular Caribbean, feeding mainly on different avian species (51%), but also on a high number of humans [39]. It is interesting to note the specimen of *Ae. albopictus*, which fed on pigs. This species feeds primarily on humans, other mammals, and birds, and less commonly on other hosts, such as reptiles, amphibians, and fish [40,41]. Other collected species, such as *Ae. scapularis* and *Psorophora* sp., were also predominant, and feed on a wide variety of avian and mammalian hosts [41].

Though host–range interactions for most *Culicoides* species in Europe have been relatively well studied [42], so far, Neotropical *Culicoides* midges have been studied very little [43]. In the present investigation, a high number of *Culicoides* midges were collected in the traps located near livestock farms, with free access to the blood of domestic animals, compared with both sylvatic habitats and poultry habitats. The presence of permanent and available domestic hosts might explain the higher diversity in accordance with other studies, as *Culicoides* density increases as the host availability increases [44]. DNA barcoding data indicated a strong affinity of *Culicoides* to feed on *Bos taurus*, which is in agreement with the preliminary results recorded in *Culicoides* spp. in the Dominican Republic [19]. These data are quite surprising, considering the large number of other mammals and birds available in the vicinity of the sampling sites. Our results might indicate that both *C. insignis* and *C. pusillus* are strictly mammophilic species. *Culicoides insignis*, the most commonly trapped species in our study, is a widespread species often associated with farm environments, and has been confirmed as the main BTV vector in the Caribbean [45,46,47,48]. Similarly, although with less information available, *C. pusillus* is also a possible BTV vector [46], commonly found in farm holdings. Both species are often trapped in association with cattle and pigs in pasture environments [49,50], and to a lesser extent, in poultry environments [51,52].

A few other interesting Diptera species were also accidentally collected by UV-suction traps. The two species of simulids trapped in our study have recently been recorded affecting outdoor activities, and, in particular, *Simulium quadrivittatum* was reported to be the predominant anthropophilic species in the proximity of flowing water courses of La Vega Province (Dominican Republic) [53]. It is worth mentioning that the traps employed are not a suitable method for capturing black flies, as they are mostly attracted by CO_2_ traps, human landing, and/or by visual attraction with colour traps [54]. Although the Dominican Republic is free of onchocerciasis, the great density of black flies found in the proximity of some water courses justifies the implementation of specific control measures. On the other hand, a few specimens of *H. irritans*, commonly known as the horn fly, were trapped inside a cow barn on a mountainside. *Haematobia irritans* is considered one of the most troublesome species within bovine production systems, due to the intense stress it imposes on the animals; however, this blood-sucking fly has been poorly recorded in the Dominican Republic [55]. Interestingly, no sand flies were captured in our study, even though CDC light traps are effective tools for the surveillance of this family [56]. *Lutzomyia cayennensis* (Floch and Abonnenc, 1941) and *Lutzomyia christophei* (Fairchild and Trapido, 1950) were collected in the early 1980s by Johnson [57]. Since then, there has been no evidence of their existence in the country, even though *Leishmania waltoni* (Shaw et al., 2015) (a member of the *Leishmania mexicana* complex), the parasite responsible for diffuse cutaneous leishmaniasis, is present in the Dominican Republic [58].

## 4. Materials and Methods

### 4.1. Study Area

The study was conducted in the municipality of Jarabacoa (19°06′29.25″ N, 70°39′00.34″ W), La Vega Province (Dominican Republic). Jarabacoa is a town of ca. 72,000 inhabitants located in the heart of the Central Cordillera, with great economic and social development, offering ecological and adventure tourism as attractions [59]. This Caribbean municipality is characterised by a tropical rainforest climate according to the Köppen climate classification [60], with an average annual temperature of 22 °C, with abundant rain through most of the year (average precipitation of 2000 mm). Due to its location and elevation (an average elevation of 529 m above sea level [a.s.l.]), the temperature varies depending on the season.

### 4.2. Entomological Survey

CDC-miniature traps equipped with UV light (BioQuip Products Inc., Rancho Dominguez, CA, USA) were operated from sunset to early morning for a minimum of 12 h. Collection jars containing insects were stored at −20 °C until further processing. The sampling sites were allocated within three categories: livestock habitats (*n* = 10), poultry habitats (*n* = 10), and sylvatic habitats (*n* = 10). Livestock habitats were farming holdings composed of domestic animals, such as cows, pigs, horses, goats, dogs, and sheep. Poultry habitats were peridomestic settings composed of pigeons, chickens, goose, and guinea fowls. Sylvatic habitats were pristine settings with little human intervention, such as banana plantations, mixed forests, pastures, and riverbanks. Trapping was carried out once in each of the 30 sampling sites from 22 March to 2 April 2021. Excluding the weekend, three traps were run each day, corresponding with each of the three habitats, thus completing the trapping in 10 days (10 days × 3 habitats = 30 collections). The weather conditions over the trapping period were constant (average diurnal T° = 22.5 °C, and average nocturnal T° = 17.4 °C, without precipitations during the night period). Sampling sites were recruited door-to-door after getting permission of the owners and/or the corresponding authorities.

### 4.3. Morphological Species Identification

Haematophagous Diptera were separated from other insects, enumerated, sorted into families, and identified morphologically at the species-level following published taxonomic keys [34,53,61,62]. The terminalia of each male mosquito was mounted on Canadian Balsam and observed under a compound microscope. Rare or little-known species of *Culicoides* females were also mounted, and diagnostic features were photographed with a Leica S9 stereomicroscope coupled to a digital system.

### 4.4. Molecular Species Identification

Damaged or unconclusive specimens were analysed by COI-based DNA barcoding (mosquitoes, *n* = 18; *Culicoides* sp., *n* = 11; *Haematobia* sp., *n* = 1) to generate COI sequences. Genomic DNA (gDNA) from the insect legs of each specimen (or whole specimens in *Culicoides*) was extracted using a NZY tissue gDNA kit (NZYTech, Lisbon, Portugal) following the manufacturer’s instructions. Maceration of the tissues was carried out manually with a sterilised piston pellet. The 658 bp region flanking of the mitochondrial COI gene was amplified by polymerase chain reaction (PCR) using the primer set LCO1490 and HCO2198, following PCR protocols described by Folmer et al. [63].

### 4.5. Identification of Host Blood Meal Sources

Blood-engorged female mosquitoes and *Culicoides* midges were separated for additional analysis. Universal vertebrate-specific primers (cytB1-F: forward 5′-CCATCMAACATCTCAGCATGATGAAA-3′ and cytB2-R: reverse 5′-GCHCCTCAGAATGATATTTGTCCTCA-3′) were used to amplify the 350 bp segment of the host mitochondrial cytochrome *b* gene (*cyt-b*) already described previously [64]. PCR was carried out in a 25 µL final volume using NZYTaq 2× Green Master Mix (NZYTech), 0.4µM of each primer, and 2 µL of template DNA.

### 4.6. Analysis of Sequences and Statistical Analysis

Amplified PCR products were purified and sequenced at STAB Vida (Monte da Caparica, Portugal) in both senses using the same primers as for PCR. Chromatogram inspection and the assembly of forward and reverse sequences edition was carried out by BioEdit Sequence Alignment Editor (version 7.2.5, Carlsbad, CA, USA). The identity at the species-level was assessed based on the analysis of the generated cox1 sequences, considering both the higher similarity in the BOLD Systems identification tool [65] and the results of homology searches from the sequences available in GenBank [66]. The respective nucleotide sequences obtained were deposited in the DNA Data Bank of Japan [67]. The identity of blood meals was assessed based on the analysis of *cyt-b* sequences, taking into account the closest database matches at the species-level of vertebrate hosts (identity ≥ 99%). Statistical analysis (the Kruskal–Wallis [KW] test) was carried to compare the abundance of *Culicoides* and mosquitoes across the different habitats using IBM SPSS Statistics for Windows (Version 27.0, Armonk, NY, USA). The Kolmogorov–Smirnov (Lilliefors modification) and Shapiro–Wilk tests were used to test for normality, and Levene’s test was used to assess the homogeneity of variance. Due to the lack of normality of the data, large standard deviations, and a lack of homogeneity of variance, non-parametric tests were employed [68]. Significant differences were considered when *p* ≤ 0.05.

### 4.7. Evolutionary Analysis by the Maximum Likelihood Method

Available *cox*1 mtDNA sequences with >600 bp of mosquito species present in the Caribbean and nearby locations were retrieved from GenBank and BOLD Systems; sequences with potential errors (flagged sequences and incorrect identifications) were excluded. Multiple alignments were carried out with MAFFT version 7 [69]. For maximum likelihood (ML) phylogenetic analyses, the choice of the best-fitting evolutionary model was based on those defined using JModeltest2 [70] on the basis of the Akaike information criterion. Tree reconstruction was carried out with Mega 11 [71]. The evolutionary history was inferred by using the ML method and the General Time Reversible model [72]. Initial tree(s) for the heuristic search were obtained automatically by applying the Neighbor-Join and BioNJ algorithms to a pairwise distances matrix estimated using the maximum composite likelihood (MCL) approach, and then selecting the topology with a higher log likelihood value. Bootstrap coefficients were calculated for 1000 replicates, and only those with >75% support are shown in the tree. The phylogenetic trees were manipulated for display using FigTree v.1.4.2 [73].

The evolutionary pairwise divergence among all *C. foxi* sequences between and within the defined groups was estimated using the Tamura 3-parameter model [74]. The haplotype diversity (Hd) and nucleotide diversity (Pi) parameters were estimated with the program DnaSP v.6.12. [75]. Haplotype networks were illustrated with a median-joining network (MJN) algorithm (ε = 0) using the software PopART v. 1.7 [76] to analyse haplotype genealogy.

## 5. Conclusions

Our study provides new insights about the fauna and host interactions of adult mosquitoes and *Culicoides* in a touristic region of the Dominican Republic. New COI-based DNA barcoding sequences of some Diptera species are provided for the first time, improving the database library of DNA barcodes, and helping to identify unknown organisms. However, the usefulness of molecular-genetic analysis and DNA markers as a tool for identification at the species-level is hampered by the limited availability of DNA barcode sequences of Neotropical mosquito species in public repositories, which has affected the accurate identification of some of the mosquito species collected. Mosquito species such as *Ae. vittatus*, *Ae. albopictus*, *Ae. scapularis*, *An. albimanus*, *Ps. confinnis*, *Cx. quinquefasciatus*, and *Cx. salinarius*, among others, are potential vectors of human pathogens, and they have been found across different types of Dominican habitats. *Culicoides insignis*, the most predominant *Culicoides* species, is considered a proven vector of BTV. The Diptera species collected showed a strong affinity to feed on domestic animals, particularly, pigs, cows, and chickens. This result contributes to deepening the knowledge on the transmission cycles of the pathogens of human and veterinary concern in the insular Caribbean.

## Figures and Tables

**Table 1:** Summary of host-derived blood meals in mosquitoes and *Culicoides* midges collected in Jarabacoa (Dominican Republic) during 2021.

Diptera Species	Number Analysed	Number Amplified (%) ^1^	Host
*Culex quinquefasciatus*	1	1 (100)	*Gallus gallus*
Culex nigripalpus *	1	1 (100)	*Gallus gallus*
*Aedes albopictus*	1	1 (100)	*Sus scrofa*
*Aedes scapularis*	1	1 (100)	*Sus scrofa*
*Psorophora confinnis*	1	1 (100)	*Sus scrofa*
*Culicoides insignis*	30	22 (73.3)	*Bos taurus*
*Culicoides pusillus*	12	5 (41.6)	*Bos taurus*
Culicoides foxi *	1	1 (100)	*Bos taurus*
Total	48	33 (68.7)	

^1^ Percentage calculated as (the number of the blood-fed specimens analysed/the number of blood-fed specimens with amplification host) × 100. * Confirmed by DNA barcoding (accession numbers LC704468 and LC704477, respectively).

## Data Availability

Not applicable.

## Supplementary Materials

The following supporting information can be downloaded at: https://www.mdpi.com/article/10.3390/pathogens11070714/s1, Table S1: Estimates of evolutionary divergence of *cox*1 over sequence pairs between groups.
