# Supplementary material for: Species Diversity, Habitat Distribution, and Blood Meal Analysis of Haematophagous Dipterans Collected by CDC-UV Light Traps in the Dominican Republic
Source: Pathogens. 2022 Jun 21;11(7):714. doi: 10.3390/pathogens11070714 (PMC9319014; doi:10.3390/pathogens11070714)
Supplement: Supplementary file 1 [file pathogens-11-00714-s001.zip › pathogens-1758365-supplementary.pdf]

Table S1

Estimates of evolutionary divergence of cox1 over sequence pairs between groups. Analyses were conducted using the Tamura 3-parameter model (Tamura, 1992). Standard error estimate(s) are shown above the diagonal and were obtained by a bootstrap procedure (1000 replicates). This analysis involved 9 nucleotide sequences with C. imicola as outgroup. Codon positions included were 1st+2nd+3rd+Noncoding. All positions containing gaps and missing data were eliminated (complete deletion option). There were a total of 614 positions in the final dataset. Evolutionary analyses were conducted in MEGA11 (Tamura et al., 2021).

	C. foxi (LC704477) D.R.	*C. foxi* (LC704478) D.R.	*C. foxi* (LC704479) D.R.	*C. foxi* (LC704480) D.R.	*C. foxi* (LC704481) D.R.	*C. foxi* (LC704482) D.R.	*C. foxi* (PLECH1418-20) C.R.	*C. foxi* (PLJAY474-20) C.R.	*C. imicola* (KT307823) (outgroup)
C. foxi (LC704477) D.R.		0,00233	0,00000	0,00000	0,00163	0,00000	0,00166	0,00228	0,01767
C. foxi (LC704478) D.R.	0,00327		0,00233	0,00233	0,00284	0,00233	0,00162	0,00327	0,01787
C. foxi (LC704479) D.R.	0,00000	0,00327		0,00000	0,00163	0,00000	0,00166	0,00228	0,01767
C. foxi (LC704480) D.R.	0,00000	0,00327	0,00000		0,00163	0,00000	0,00166	0,00228	0,01767
C. foxi (LC704481) D.R.	0,00163	0,00491	0,00163	0,00163		0,00163	0,00231	0,00162	0,01786
C. foxi (LC704482) D.R.	0,00000	0,00327	0,00000	0,00000	0,00163		0,00166	0,00228	0,01767
C. foxi (PLECH1418-20) C.R.	0,00163	0,00163	0,00163	0,00163	0,00327	0,00163		0,00283	0,01768
C. foxi (PLJAY474-20) C.R.	0,00327	0,00656	0,00327	0,00327	0,00163	0,00327	0,00491		0,01802
C. imicola (KT307823) (outgroup)	0,17669	0,17885	0,17669	0,17669	0,17883	0,17669	0,17670	0,18096	

D.R.= Dominican Republic, R.C.= Costa Rica
